# *Verticillium dahliae* PevD1, an Alt a 1-like protein, targets cotton PR5-like protein and promotes fungal infection

**DOI:** 10.1093/jxb/ery351

**Published:** 2018-10-05

**Authors:** Yi Zhang, Yuhan Gao, Yingbo Liang, Yijie Dong, Xiufen Yang, Dewen Qiu

**Affiliations:** 1 The State Key Laboratory for Biology of Plant Disease and Insect Pests, Institute of Plant Protection, Chinese Academy of Agricultural Sciences, Beijing, China; 2 Key Laboratory of Control of Biological Hazard Factors (Plant Origin) for Agri-product Quality and Safety, Ministry of Agriculture, Institute of Plant Protection, Chinese Academy of Agricultural Sciences, Beijing, China; 3 Key Laboratory of Integrated Pest Management in Crops, Ministry of Agriculture, Institute of Plant Protection, Chinese Academy of Agricultural Sciences, Beijing, China

**Keywords:** Alt a 1, *Gossypium hirsutum*, interaction, pathogenesis-related protein 5 (PR5), PevD1, *Verticillium dahliae*

## Abstract

Alt a 1 family proteins (AA1s) have only been observed in the Dothideomycetes and Sordariomycetes classes of fungi, and their biological functions have remained poorly understood. *Verticillium dahliae*, a soil-borne pathogen that causes plant wilt disease, secretes hundreds of proteins during the process of pathogenic infection, including the AA1 member PevD1. In this study, we found that the *pevd1* transcript was present in all of the hosts studied (cotton, Arabidopsis, tomato, and tobacco) and showed elevated expression throughout the infection process. Furthermore, *pevd1* knockout mutants displayed attenuated pathogenicity compared with the wild-type (WT) strain and complemented strains in hosts. A partner protein of PevD1, pathogenesis-related protein 5 (PR5)-like protein GhPR5, was isolated from cotton (*Gossypium hirsutum*) plants by co-purification assays, and the PevD1–GhPR5 interaction was determined to be localized in the C-terminus (PevD1b, amino acids residues 113–155) by pull-down and yeast two-hybrid techniques. Re-introduction of the *pevd1b* gene into a *pevd1* knockout mutant resulted in restoration of the virulence phenotype to WT levels. In addition, PevD1b, which is similar to PevD1, decreased the antifungal activity of GhPR5 *in vitro*. Our findings reveal an infection strategy in which *V. dahliae* secretes PevD1 to inhibit GhPR5 antifungal activity in order to overcome the host defence system.

## Teaser

PevD1, an Aa1-like protein secreted by *Verticillium dahlia,* promotes infection by inhibiting the antifungal activity of *Gossypium hirsutum* PR5. .

## Introduction

Plants have evolved two major general defence systems to respond to attacks by pathogens. The primary defence system is that plants respond to pathogen-associated molecular patterns (PAMPs) via pattern recognition receptors (PRRs), a process termed PAMP-triggered immunity (PTI) (Jones and Dangl, 2006). PTI causes rapid defence reactions, such as ion fluxes, cell wall reinforcement, the production of reactive oxygen species (ROS), and the expression of defence-related genes (Zipfel, 2008, 2009). However, successful pathogens can interfere with PTI by delivering effectors, evading recognition, or suppressing signalling pathways. In turn, the hosts use resistance proteins to recognize the effectors, leading to the secondary defence system of effector-triggered immunity (ETI) (Jones and Dangl, 2006; Zipfel, 2008). ETI commonly results in local cell death, known as the hypersensitive response (HR).

During the recognition of pathogens, most of the inducible defence-related proteins are associated with pathogenesis-related proteins (PRs), which have been divided into 17 families and play important roles in plant defence systems (van Loon *et al.*, 2006). Of these, the PR5 family proteins, which are also referred to as thaumatin-like proteins, have been reported in many species upon infection with pathogens and have been associated with antimicrobial activity. Some PR5s have been shown to have antifungal activity *in vitro* against *Verticillium dahliae* (Abad *et al.*, 1996; Pressey, 1997; Min *et al.*, 2004; Tjamos *et al.*, 2005; van Loon *et al.*, 2006). The tobacco PR5 protein osmotin is inducible by both osmotic stress and pathogens, and its homologs in tomato and potato have been shown to possess anti-oomycete activity *in vitro* against *Phytophthora infestans*, and overexpression of tobacco PR5 in tobacco and potato plants can enhance disease resistance against this pathogen. ATLP3, a PR5 protein identified from Arabidopsis, displays *in vitro* antifungal activity against *V. dahliae*, *V. alboatrum*, and *Fusarium oxysporum* (Hu and Reddy, 1997). Overexpression of a rice thaumatin-like protein results in an enhanced resistance to *Alternaria alternata* in tobacco (Velazhahan and Muthukrishnan, 2004).

*Verticillium dahliae*, which is the causal agent of cotton wilt disease, is a pathogenic fungus with a very wide host range of over 200 dicotyledonous plant species (Fradin and Thomma, 2006). It produces various effector molecules to modulate its infection, although not much is known regarding the components that are crucial for its pathogenicity. In general, cell wall-degrading enzymes (CWDEs), toxins, and elicitor-like molecules have been the focus of most studies. *Verticillium dahliae* secretes CWDEs that act as a virulence factor in its interactions with plants, among which pectinolytic enzymes are the best-characterized and are thought to have important roles in pathogenicity (Dobinson *et al.*, 2004). Some of these enzymes have been shown to cause wilt symptoms and promote the necrosis of plant tissues (Cooper and Wood, 1980). In a recent study, a *V. dahliae* pectate lyase, VdPEL1, was shown to induce plant cell death and contribute to virulence (Yang *et al.*, 2018). Several CWDEs, such as ethylene-inducing xylanase (EIX) (Enkerli *et al.*, 1999) and xyloglucan-specific endoglucanase (XEG1) (Ma *et al.*, 2015), have also been recognized as PAMPs. In addition, *V. dahliae* produces phytotoxins and elicitor proteins that induce host cell death (Pegg, 1965; Wang *et al.*, 2012), suggesting that they may also be associated with pathogenicity. A high-molecular-weight protein–lipopolysaccharide (PLP) complex has been reported to be associated with disease symptoms in susceptible host plants and to be required for pathogenicity (Buchner *et al.*, 1982). Furthermore, many elicitor-like proteins isolated from *V. dahliae*, such as PevD1 (Wang *et al.*, 2012), VdCP1 (Zhang *et al*., 2017), and VdNEP (Wang *et al.*, 2004), can induce plant defence responses and cell death.

The Alt a 1 family of proteins (AA1s; Pfam family PF16541) are only present in the Dothideomycetes and Sordariomycetes classes of fungi (Chruszcz *et al.*, 2012). The AA1 produced by the first member ‘Alt a 1 (Aa1)’, identified from *A*. *alternata*, is the source of the name of this protein family (Cramer and Lawrence, 2003). Studies have shown that Aa1 is involved in *Alternaria* spore germination and that it is localized exclusively in the spore cell wall (Mitakakis *et al.*, 2001; Twaroch *et al.*, 2012). Alt b 1 (Ab1), a homolog of Aa1, has been identified from *A. brassicicola*. High expression of *Ab1* mRNA has been detected during the infection process of Arabidopsis (Cramer and Lawrence, 2004). An AA1 member from *Magnaporthe oryzae* has been demonstrated to bind to the plant plasma membrane and to induce necrosis, the accumulation of ROS, and the expression of several defence-related genes, suggesting that it has a PAMP role (Zhang *et al.*, 2017*b*). To date, numerous *AA1* genes have been identified from over 70 species, although the biological functions of AA1 proteins with respect to plant–fungal interactions remain unclear.

PevD1 has previously been isolated from *V. dahliae* culture filtrates and shown to exhibit an elicitor-like activity by inducing defence responses in tobacco plants (Wang *et al.*, 2012). PevD1-induced disease resistance in tobacco has been demonstrated to be modulated by an asparagine-rich protein, NbNRP1 (Liang *et al.*, 2018). Interestingly, overexpression of PevD1 in Arabidopsis has been reported to promote early flowering by interacting with AtNRP1 and plants infected with *V. dahliae* flower earlier than uninfected plants (Zhou *et al.*, 2017), implying that PevD1 has a role in mediating plant development during *V. dahliae* infection. However, even though it is one of the most abundant proteins among the many secreted by *V. dahliae*, the biological role of PevD1 in the infection process, and particularly its possible function in pathogenicity, remains unclear. In this study, we determined that PevD1 is an Aa1-like protein, and an interacting protein named GhPR5 was identified from cotton plants. Our findings show that *V. dahliae* secretes PevD1 to inhibit the antifungal activity of GhPR5 as a strategy to resist the plant defence system and to promote fungal infection.

## Materials and methods

### Plant material, culturing of strains, inoculation, and pathogenicity assays

Plants of cotton (*Gossypium hirsutum*), *Arabidopsis thaliana*, tobacco (*Nicotiana benthamiana*), and tomato (*Solanum lycopersicum*) were grown as previously described (Frías *et al.*, 2011; Wang *et al.*, 2012; Zhang *et al.*, 2015). Strains of *Verticillium dahliae* and *Botrytis cinerea* were grown on potato dextrose agar (PDA) medium at 25 °C, and *V*. *dahliae* was cultured in liquid Czapek Dox medium for 5 d at 25 °C for spore production (Wang *et al.*, 2012; Zhang *et al.*, 2015). Ungerminated conidia were used as controls in fungal gene expression analysis. *Agrobacterium tumefaciens* AGL-1 was cultured on liquid LB medium (rifampicin 50 μg ml^–1^) at 28 °C (Liu *et al.*, 2013).

Pathogenicity assays for *V. dahliae* strains were conducted using 2-week-old cotton plants of cv. Guoxin (more tolerant) and Chuangxin (less tolerant), or 4-week-old Arabidopsis, tobacco, and tomato seedlings (Zhang *et al.*, 2017*a*). For each strain, conidial suspensions at 5 × 10^6^ ml^–1^ in sterile water were prepared. Plant seedlings were grown in soil in individual pots, and 15 pots were prepared for each strain, with this set-up replicated three times. Samples of the conidial suspension (15 ml) were aliquoted into 15 separate trays, and each pot was placed in a tray until complete absorption of the liquid had occurred. The inoculated seedlings were then grown for 2–3 weeks at 28 °C, with a day/night period of 16/8 h, and disease symptoms were observed at 21 d post-inoculation on cotton or 15 d post-inoculation on tobacco and tomato. Vascular discoloration was observed by cutting the shoots at 21 d post-inoculation on cotton. For fungal biomass quantification, roots of three inoculated plants were harvested at 21 d after inoculation and ground to powder for genomic DNA extraction. The fungal biomass was determined by qPCR as described previously (Gui *et al.*, 2018), with three biological replicates, and triplicate reactions were performed for each of the biological replicates. *Verticillium* EF-1α was used to quantify fungal colonization, and the cotton *18S* gene served as the internal control (Supplementary Table S1 at *JXB* online).

### Quantitative real-time PCR

Roots of host plants (2-week-old cotton and 4-week-old Arabidopsis, tobacco, and tomato) were inoculated with a spore suspension as described above. At the same time, spore suspensions were spread on plates containing PDA medium, covered with cellophane, and then cultured. Subsequently, samples (100 mg of roots or the cellophane covering the plates) were collected at the indicated time points to assess the expression levels of *pevd1*. Total RNA from *V. dahliae* and *V. dahliae*-inoculated plants was extracted with an EZNA^®^ Fungal RNA Kit (R6840, Omega) and plant RNA kits (ET121 and ER301, TransGen Biotech), respectively. First-strand cDNA was synthesized from the extracted RNA using a cDNA Synthesis kit (AT341, TransGen Biotech). The synthesized cDNA was then used as a template for qPCR, which was performed under the following conditions: an initial 50 °C incubation step for 2 min and a 94 °C denaturation step for 10 min, followed by 40 cycles of 94 °C for 5 s and 60 °C for 34 s. Amplification was performed using an ABI7500 Real-Time PCR system with a qPCR kit (AQ111, TransGen Biotech). *Verticillium* elongation factor 1-a (EF-1α), the cotton *18S* gene, Arabidopsis *Actin2* gene, tobacco EF-1α, and tomato *Actin* gene were used as internal references to normalize the amount of RNA in different reactions (for primers see Supplementary Table S1). For each gene, qPCR assays were repeated three times, each with three biological replicates. Relative mRNA quantities were calculated from the average values using the ΔΔ*C*_T_ method (Schmittgen and Livak, 2008).

### Deletion and complementation of *pevd1* and its truncated fragments

*Verticillium dahliae* mutant (*Δpevd1*) and complemented strains (*Δpevd1*-Res, *Δpevd1*-Res1a and *Δpevd1*-Res1b) were constructed using an *A. tumefaciens*-mediated transformation method (Liu *et al.*, 2013). All primers used are shown in Supplementary Table S1. The *Agrobacterium* strain AGL-1 was used to transform the recombinant plasmids into *V. dahliae* spores.

### Expression and purification of proteins and preparation of polyclonal antibodies

PevD1 (or its truncated fragments) without the predicted signal peptide (SP) and termination codon was inserted into the *Eco*RI and *Xba*I sites of the plasmid pPICZαA. The recombinant plasmid pPICZαA-*pevd1* was linearized with the restriction enzyme *Pme*I and was subsequently transformed into *Pichia pastoris* KM71H for expression (Zhang *et al.*, 2017*b*). The *GhPR5* gene without the predicted SP and the vacuole-targeting sequence was inserted into the *Bam*HI and *Xho*I sites of the plasmid pRSETA (Invitrogen). The recombinant plasmid pRSETA-*GhPR5* was transformed into *E. coli* BL21(DE3)pLysS for expression. The recombinant GhPR5 protein was expressed in inclusion bodies, and the purification and refolding of GhPR5 was carried out as previously described (González *et al.*, 2017; Zhang *et al.*, 2017*c*). Proteins were detected by SDS-PAGE (RTD6110, Real-Times, Beijing), and were frozen at –80 °C in small aliquots until use. The protein concentration was measured using a BCA™ Protein Assay Kit (DQ111, TransGen Biotech). All primers used are shown in Supplementary Table S1. The purified GhPR5 was used as an antigen for antibody production. Antibodies were produced in rabbits by the HuaAn Biotechnology Company.

### Treatment of *V. dahliae* strains with GhPR5

The *V. dahliae* strains WT, *Δpevd1*, *Δpevd1*-Res, *Δpevd1*-Res1a, and *Δpevd1*-Res1b were tested for their response to GhPR5. Following the method of previous studies (Sella *et al.*, 2014; Quarantin *et al.*, 2016), microtiter plate wells were filled with 200 μl potato dextrose broth (PDB, pH 6.85) containing purified GhPR5 (2 μM) or GhPR5 plus PevD1 (or plus PevD1a/PevD1b, 2 μM). Controls were carried out with buffer instead of the protein solution. A final concentration of 10^6^ ml^–1^ spores of *V. dahliae* strains were added into the PDB, and 68 μg ml^–1^ of resazurin dye (R7017, Sigma-Aldrich) was added to each well to measure fungal growth. The plates were incubated at 25 °C in the dark and the absorbance was measured at 578 nm over a time-course to evaluate *V. dahliae* growth inhibition. The same experiment was also carried out to assess the activity of refolding GhPR5, with 0.2 U of β-1,3-glucanase (67138, Sigma-Aldrich) and BSA (2 μM) used as positive and negative controls, respectively. Measurements were repeated at least twice, each with three biological replicates. In addition, *V. dahliae* spores treated with GhPR5 or GhPR5 plus PevD1 (or PevD1a/PevD1b) for 12 h were used for pathogenicity assays as described above.

### Yeast two-hybrid assays

To confirm the protein interactions, experiments were performed using the Matchmaker™ Gold Yeast Two-Hybrid (Y2H) System as described previously (Zhou *et al.*, 2017). The coding sequences of PevD1 (without the SP or its truncated fragments) and GhPR5 (without the SP) were cloned into the bait vector pGBKT7 (BD-*pevd1*) and the prey vector pGADT7 (AD-*GhPR5*), respectively. The bait and prey vectors were co-transformed into the Gold Yeast strain, and the subsequent assays were performed according to the manufacturer’s instructions. The transformants were selected on synthetic dextrose medium without Trp and Leu (SD/–Trp/–Leu; double dropout, DDO). Interaction were indicated by the ability of transformants to grow on SD medium lacking Trp, Leu, His, and Ade (SD/–Trp/–Leu/–His/–Ade; quadruple dropout, QDO).

### Transient expression, pull-down assays, and western blot analysis

The genes *pevd1* and *pevd1*-*ΔSP* were inserted into the *Eco*RI and *Kpn*I sites of plasmid pYBA1132, which contains a GFP tag (green fluorescent protein; Yan *et al.*, 2012). *Agrobacterium* (GV3101)-mediated transient expression was performed using leaf infiltration as described previously (Kettles *et al.*, 2017), and this experiment was performed three times with at least six leaves infiltrated each time. Proteins were subsequently extracted from tobacco leaves as previously described (Zhu *et al.*, 2017).

For pull-down assays, 10 g of roots or leaves from 2-week-old cotton inoculated with *V. dahliae* spore suspension were collected at 4-d post-inoculation, then frozen with liquid nitrogen and ground in a mortar. Then, 35 ml of protein extraction buffer [50 mM Tris, 200 mM NaCl, 1% Triton X-100, 1 mM PMSF, 0.2% protease inhibitor cocktail (P9599, Sigma), pH 8.0] was added to the tissue powder, and the mixture was transferred to a 50-ml tube and incubated at 4 °C for 2 h with slight shaking. After centrifuging at 5000 *g* for 5 min, the supernatant was passed through filter paper and then concentrated by using a 3-kD ultrafiltration device. The protein concentrate was mixed with PevD1 (or two different proteins were mixed) and incubated for 2 h at 4 °C with slight shaking, then 20 μl Anti-HA Affinity Gel beads (E6779, Sigma-Aldrich) was added into the protein mixture and the manufacturer’s instructions were followed. Proteins were separated by SDS-PAGE and transferred onto membranes (PVDF), after which the blots were analysed using the appropriate antibodies. The pixel intensities on images of the western blots were quantified using the ImageJ software. The pull-down and western blot assays were repeated at least twice.

### Determination of the endogenous levels of salicylic acid

Cotton root tissues inoculated with either a *V. dahliae* spore suspension or water were collected at 4-d post-inoculation, weighed, and frozen in liquid nitrogen. The endogenous salicylic acid (SA) content of the samples was extracted and measured according to previous studies (Bowling *et al.*, 1994; Lv *et al.*, 2016). The assay was carried out at least three times.

### Bioinformatics analysis

Amino acid sequence alignment was performed using the MUSCLE software (http://www.drive5.com/muscle/) and processed with AL2CO (Pei and Grishin, 2001) to generate conservation indices for every amino acid. The phylogenetic tree was reconstructed with MEGA 6.0 (Tamura *et al.*, 2013) using the maximum-likelihood method. The crystal structures previously determined for the PevD1 protein (Zhou *et al.*, 2017) and predicted for the GhPR5 protein using SWISS-MODEL (https://swissmodel.expasy.org/interactive) were used. The protein structure was analysed using the PyMOL Molecular Graphics System, v.2.0, and Poisson–Boltzmann electrostatic potentials were computed with the APBS plugin in PyMOL. Protein–protein docking model structures for the PevD1–GhPR5 complex were obtained using ClusPro 2.0 (https://cluspro.bu.edu/login.php).

## Results

### PevD1 is an Alt a 1-like protein

**Fig. 1.**
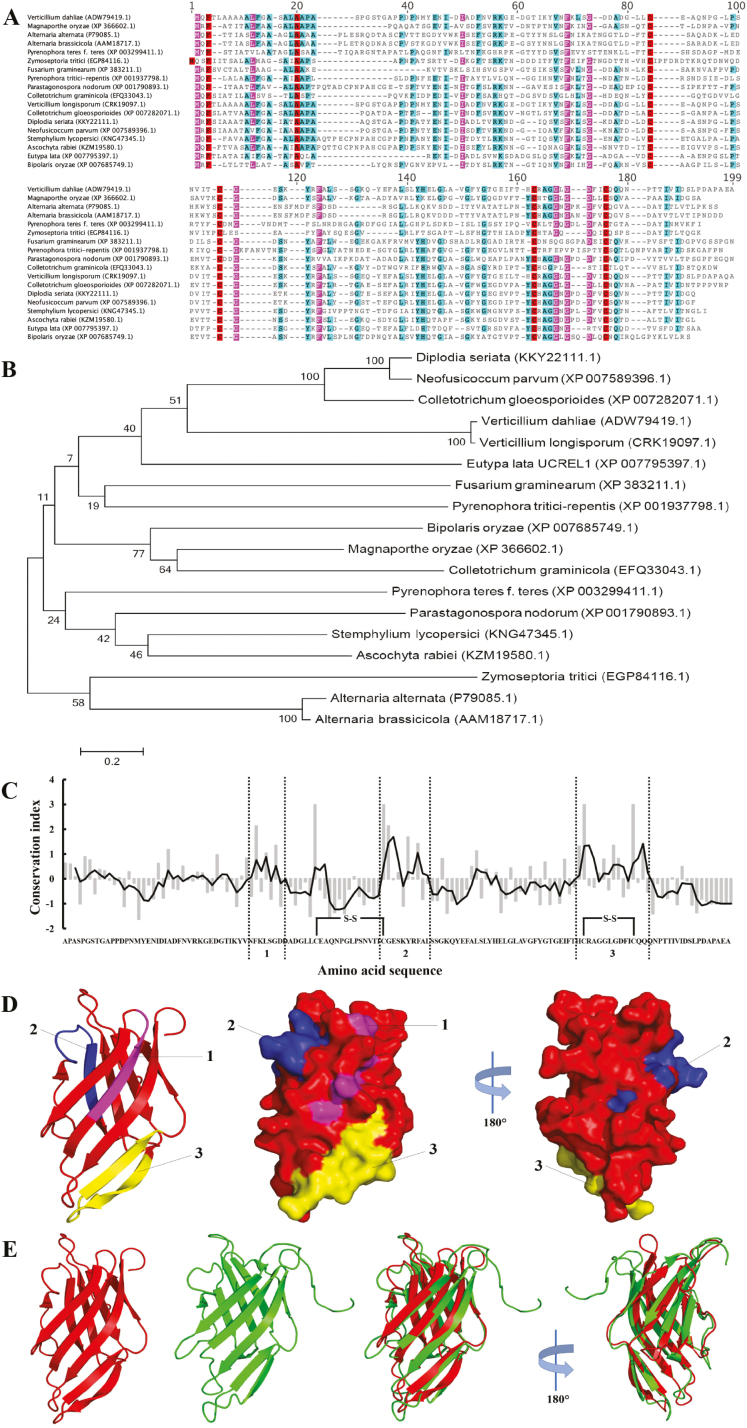
Characteristics of the Alt A 1 protein member PevD1. (A) Sequence alignment of selected PevD1 homologs was performed using MUSCLE. Identical amino acid residues are highlighted in red, similar residues are indicated by magenta (80–100% identity of amino acid residues) and light blue (60–80% identity of amino acid residue). The GenBank accession numbers of *Verticillium dahliae* PevD1 and its homologs are indicated. (B) The evolutionary relationships between PevD1 and its homologs from other fungi was determined using the maximum-likelihood algorithm. (C) Conservation indices (grey bars) were calculated using AL2CO for every PevD1 residue from the alignment of 36 complete AA1 proteins. Zero corresponds to the average conservation index for the whole protein. Regions 1–3 indicate where the conservation indices that are well above average. (D) The structure of PevD1 (PDB ID: 5XMZ) was shown as a diagram and surface views. The three conserved regions (1–3) are shown in the structure of PevD1, and correspond to magenta, blue, and yellow. (E) Structures of PevD1 (red) and Alt a 1 (green, PDB ID: 3V0R) were aligned using PyMOL.

PevD1 (VDAG_02735) has previously been identified as an abundant protein in the culture medium of *V. dahliae* (Wang *et al.*, 2012). The protein contains 155 amino acids and has a predicted SP at the cleavage site between amino acids 18 and 19 (SignalP 4.1 server; http://www.cbs.dtu.dk/services/SignalP/), indicating that PevD1 may be a secretory protein. The mature protein (without the SP) has a predicted molecular mass of 14 523.1 Da and a pI of 4.18. Our amino acid sequence analysis revealed that it has a low molecular weight, four conserved cysteine residues (C70, C84, C125, and C135), and is homologous to three characterized protein members of the AA1 family, namely Alt a 1 (Chruszcz *et al.*, 2012), Alt b 1 (Cramer and Lawrence, 2003), and MoHrip1 (Zhang *et al.*, 2017*b*), as well as several typical protein members from different species (Fig. 1A). Phylogenetic analysis indicated that homologs of PevD1 are present across a number of important plant pathogens (Fig. 1B). As AA1s are widely distributed and well conserved among the Dothideomycetes and Sordariomycetes classes of fungi, conserved protein region(s) are present in this protein family. To identify such regions in PevD1, an alignment of 36 AA1 proteins was analysed to identify regions of the sequences that are strongly conserved among these family members (Fig. 1C). The results identified three conserved regions in AA1s in the crystal structure of PevD1 (Zhou *et al.*, 2017) (Fig. 1D). PevD1 was highly similar to Aa1 (Chruszcz *et al.*, 2012) (Fig. 1E). Together, these data indicated that PevD1 is a member of AA1 family.

### PevD1 contributes to the pathogenicity of *V. dahliae*

**Fig. 2.**
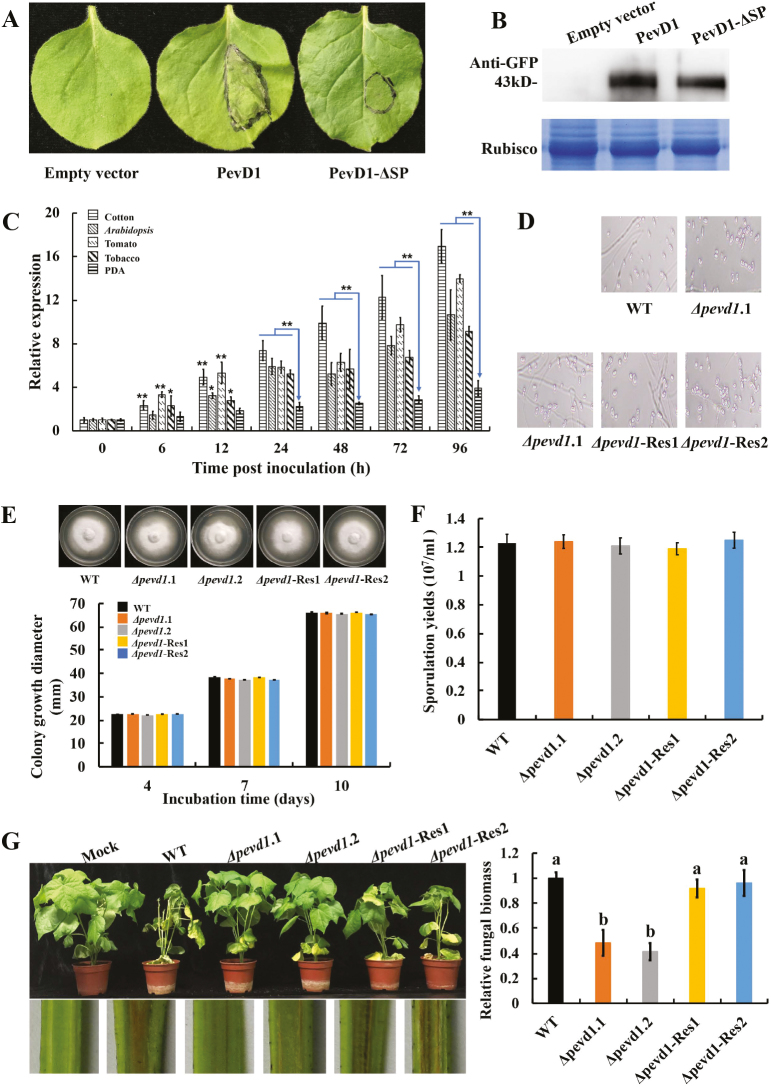
Expression patterns and biological function of PevD1. (A) PevD1-induced necrosis in *Nicotiana benthamiana*. Leaves of were infiltrated with *Agrobacterium tumefaciens* containing the empty vector pYBA1132, pYBA1132-*pevd1*, or pYBA1132-*pevd1*-ΔSP. The images were taken 3 d after agro-infiltration. (B) Western blot analysis (anti-GFP) of the transiently expressed proteins obtained from the three agro-infiltrated leaves. Coomassie brilliant blue (CBB) is shown as a total protein loading control. (C) Expression profiles of *pevd1* compared to ungerminated spores (0 h), as determined by qPCR. The expression levels of *pevd1* in *Verticillium dahliae*-inoculated plants (or PDA plates, potato dextrose agar) are shown as mRNA levels relative to those in ungerminated spores (0 h, set as 1). Data are means (±SD) of three replicates. Significant differences were determined using Student’s *t*-test: *P<0.05, **P<0.01. (D–F) The phenotypes of the *Δpevd1* and *Δpevd1*-Res strains with respect to their mycelium/spore morphology, growth rate, and sporulation capacity compared with the wild-type (WT) strain. Data are means (±SD) and each assay was replicated three times. No significant differences compared with the WT were found (Student’s *t*-test). (G) Disease symptoms in cotton plants (*G. hirsutum* cv. Chuangxin) at 21 d post-inoculation (top) and discoloration of shoot longitudinal sections (bottom), together with qPCR analysis of fungal biomass from 100 mg of ground roots, expressed relative to that of the wild-type. Data are means (±SD) of three replicates and different letters indicate significant differences as determined by ANOVA and *post hoc* Tukey’s test: *P*<0.01.

To characterize the function of PevD1 during infection, the full-length protein or PevD1-ΔSP (without SP) was transiently expressed in *N. benthamiana* by agro-infiltration. The results showed that PevD1 induced distinct necrosis in leaves while the control did not (Fig. 2A) and PevD1-ΔSP was only able to induce slight necrosis, suggesting that it was specifically induced by PevD1. Western blot analysis with anti-GFP antibodies showed that PevD1-GFP and PevD1-ΔSP-GFP were successfully expressed in plant leaves (Fig. 2B).

To investigate whether PevD1 has an important role in the interaction between *V. dahliae* and the host, a qPCR analysis was carried out to determine the mRNA levels of *pevd1* at various stages of infection in hosts and during growth on PDA medium. The mRNA levels of *pevd1* increased continually during the infection process compared to ungerminated conidia in all the hosts studied, and were significantly higher compared with the expression level observed in cells grown on PDA (Fig. 2C). These results indicated that *pevd1* was expressed during infection, especially in the later stages, and suggested that PevD1 plays an important role in the infection process. To further test this hypothesis, *Δpevd1* and *Δpevd1*-Res strains were generated (Supplementary Fig. S1). The phenotypes of these strains with respect to their mycelium/spore morphology, growth rate/colony morphology, and sporulation capacity did not differ from those of the wild-type (WT) strain (Fig. 2D–F). However, the *Δpevd1* strains exhibited reduced virulence and the *Δpevd1*-Res strains exhibited restored virulence compared with the WT strain in cotton (Fig. 2G). Quantification in cotton plants using qPCR showed that inoculation with the *Δpevd1* strains resulted in significantly less *in planta* fungal biomass compared with plants inoculated with the WT and *Δpevd1*-Res strains (Fig. 2G). In addition, tobacco and tomato plants inoculated with these *V. dahliae* strains showed similar symptom phenotypes to cotton (Supplementary Fig. S2), suggesting that PevD1 is required for full virulence of *V. dahliae* on hosts.

### Cotton PR5-like protein co-purifies with PevD1 as determined by *in vitro* affinity pull-down

**Fig. 3.**
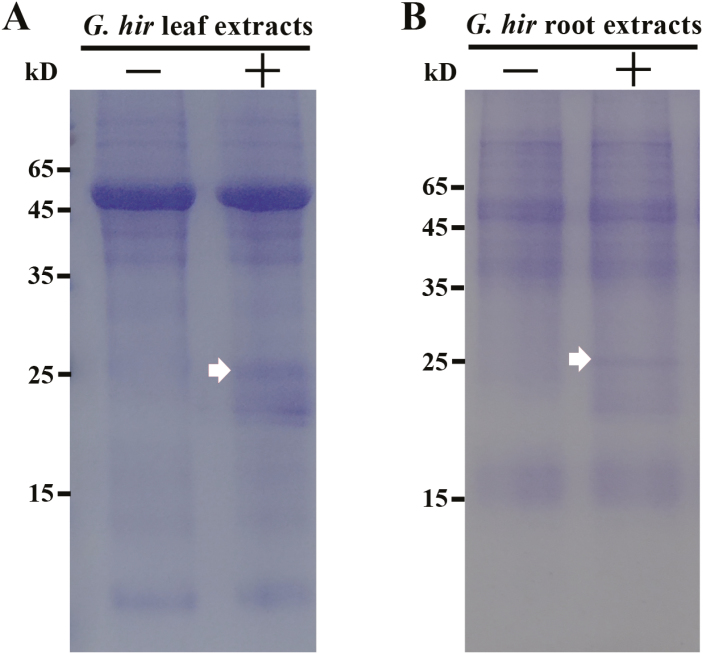
*Gossypium hirsutum* pathogenesis-related protein 5 (GhPR5) co-purifies with PevD1. (SDS-PAGE of the proteins resulting from pull-down assays with *G. hirsutum* leaf (A) or root extracts (B), incubated with (+) or without (–) PevD1.

Given PevD1 was shown to be a secretory protein that contributes to virulence, unknown target(s) for it must be present in host cells. To identify cotton protein(s) targeted by PevD1, a pull-down assay was performed. Total leaf and root proteins were extracted from cotton plants at 4 d after inoculation with *V. dahliae*. PevD1 (expressed in *P. pastoris*, Supplementary Fig. S3) was mixed with cotton leaf or root extracts, followed by purification with a HA Affinity Gel. After elution, a 25-kD protein band was co-purified with PevD1 from both cotton leaf and root extracts, whereas no additional band appeared in the control assays with extracts alone (Fig. 3). The band was cut from the gel and analysed by mass spectrometry (MS) for protein identification, which revealed a match with the *G. hirsutum* osmotin-like protein (UniProt No. Q2HPG3), which we designated as GhPR5.

**Fig. 4.**
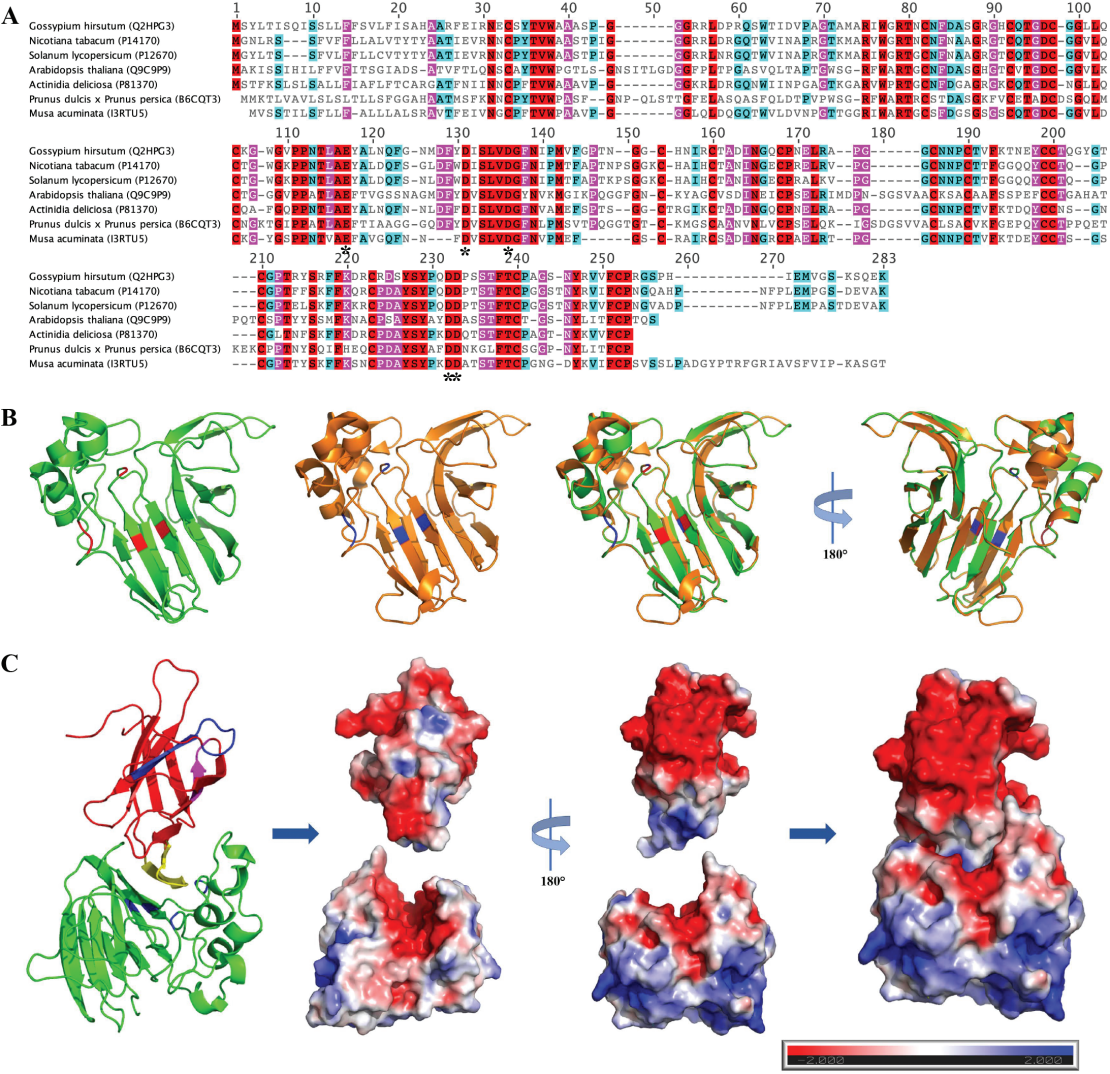
Sequence and structure analysis of GhPR5 homologs and prediction for a PevD1–GhPR5 interaction model. (A) Sequence alignment of selected GhPR5 homologs was performed using MUSCLE. Identical amino acid residues are highlighted in red, similar residues are indicated by magenta (80–100% identity of amino acid residues) and light blue (60–80% identity of amino acid residue). The GenBank accession numbers are indicated. The crucial amino acid residues of the electrostatically negative cleft of PR5s where the antifungal enzymatic activity occurs are indicated by asterisks. (B) Structures of GhPR5 (green, predicted by SWISS-MODEL) and osmotin (orange, PDB ID: 4L2J) were aligned using PyMOL. (C) The predicted PevD1–GhPR5 interaction model as determined by ClusPro 2.0. In the diagram on the left, PevD1 is coloured red, and magenta, blue, and yellow correspond to the three conserved regions. GhPR5 is coloured green. The structures to the right show the Poisson–Boltzmann electrostatic potential mapped onto the molecular surface of the PevD1–GhPR5 complex. The scale bar refers to electrostatic potential values in units of k*T* per unit charge (k, Boltzmann’s constant; *T*, absolute temperature).

Osmotin, named on the basis of its induction by osmotic stress (Singh *et al.*, 1985), belongs to the PR5 family. Osmotins and other PR5s have been shown to exhibit antifungal activity against a broad range of plant pathogens (Abad *et al.*, 1996; Min *et al.*, 2004; Tjamos *et al.*, 2005; van Loon *et al.*, 2006; Anil Kumar *et al.*, 2015). GhPR5, an osmotin-like protein, contains 242 amino acids and has a predicted N-terminal SP to secrete it into the extracellular space and a vacuole-targeting C-terminal signal to transport it to vacuoles. The amino acid sequence of GhPR5 exhibited five conserved amino acid residues that were also present in the antifungal enzymatic-activity region of PR5s, showing that it is homologous to PR5s (Fig. 4A). The structure of GhPR5 exhibited the highest identification score with osmotin from tobacco (Fig. 4B). Protein–protein docking calculations were carried out using the experimental X-ray structure of PevD1 (PDB ID: 5XMZ) and the predicted structure of GhPR5. The best complex candidates indicated the formation of PevD1–GhPR5 aggregates in the electrostatically negative cleft of GhPR5, where the antifungal enzymatic activity occurs. The cleft was partially blocked with PevD1 facing an electrostatically positive prominent region (Fig. 4C).

### A conserved 40-amino-acid region of PevD1 is involved in its binding to GhPR5 and is sufficient for fungal virulence

**Fig. 5.**
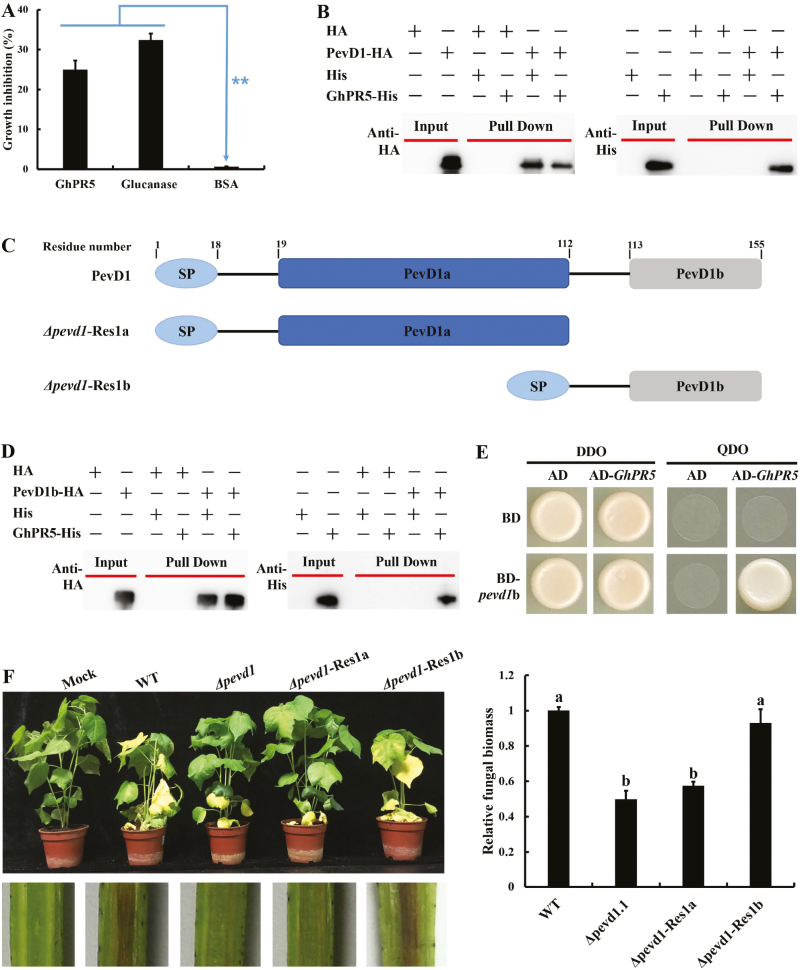
PevD1b interacts with GhPR5 and is sufficient for fungal virulence. (A) The sensitivity of the *Verticillium dahliae* wild-type (WT) strain in the presence of GhPR5. Growth was determined spectrophotometrically by measuring the absorbance of the resazurin vital dye at 578 nm. BSA at the same concentration was used as a negative control and glucanase was used as a positive control. Data are means (±SD) of three replicates and significant differences were determined using Student’s *t*-test: **P<0.01. (B) The physical interaction of PevD1 and GhPR5 *in vitro* was verified by a pull-down assay. PevD1 and a HA tag were incubated in binding buffer containing HA beads with or without GhPR5 with a His tag; the beads were then washed five times and eluted for western blot analyses. The HA and His tags did not appear due to their low molecular masses. (C) Schematic diagram of the generation of the truncated complemented mutants fused with a signal peptide (SP). (D) Verification of a physical interaction between PevD1b and GhPR5 *in vitro* by pull-down assay. The HA and His tags did not appear due to their low molecular masses. (E) Yeast two-hybrid analysis of the interaction between PevD1 and GhPR5. Double dropout (DDO, SD/–Trp/–Leu,) and quadruple dropout (QDO SD/–Trp/–Leu/–Ade/–His,) were used as non-selective and selective media, respectively. (F) Disease symptoms in cotton plants (*G. hirsutum* cv. Chuangxin) at 21 d post-inoculation (top) and discoloration of shoot longitudinal sections (bottom), together with qPCR analysis of fungal biomass from 100 mg of ground roots, expressed relative to that of the wild-type. Data are means (±SD) of three replicates and different letters indicate significant differences as determined by ANOVA and *post hoc* Tukey’s test: *P*<0.01.

Osmotins have been demonstrated to exhibit antifungal activity (Anil Kumar *et al.*, 2015; González *et al.*, 2017), but a similar activity for GhPR5 has not been reported. PR5s have only previously been expressed in *E. coli* (Sharma *et al.*, 2013; González *et al.*, 2017). Therefore, we used the pRSETA vector system to express GhPR5 lacking the N-terminal SP domain and the C-terminal vacuolar-targeting sequence, with the protein tagged (6×His) for purification and subsequent antibody recognition. In addition, the HA and His tag were expressed in *P*. *pastoris* (Supplementary Fig. S4). We found that GhPR5 was able to inhibit the growth of *V. dahliae*, as did the positive control (β-1,3-glucanase) (Fig. 5A), suggesting that GhPR5 was properly folded.

To further assess whether PevD1 could directly interact with GhPR5, we conducted *in vitro* pull-down assays using recombinant proteins. The results showed that PevD1-HA but not HA could pull down GhPR5-His (Fig. 5B). Based on the docking calculations, PevD1 may bind to GhPR5 with its β8–9 region, which contains a loop (marked in yellow in Fig. 1D). Hence, two truncated PevD1 fragments (PevD1a, β1–6; and PevD1b, β7–10; Fig. 5C) were expressed in *P. pastoris* (Supplementary Fig. S3) to evaluate the interaction between the PevD1 regions and GhPR5. Pull-down and Y2H assays suggested that PevD1b interacts with GhPR5 (Fig. 5D, E). To further determine whether PevD1b contributes to full virulence in the *V. dahliae*–plant interaction, we generated the complemented strains *Δpevd1*-Res1a (re-introduction of the *pevd1a* gene into the *pevd1* knockout mutant) and *Δpevd1*-Res1b (re-introduction of the *pevd1b* gene into the *pevd1* knockout mutant; Fig. 5C, Supplementary Fig. S5). We found that the *Δpevd1*-Res1a strain displayed decreased virulence and the *Δpevd1*-Res1b strain showed similar virulence compared with the WT strain (Fig. 5F). Quantification of fungal biomass in cotton plants as determined by qPCR showed that inoculation with the *Δpevd1*-Res1b strains resulted in significantly more *in planta* fungal biomass compared with plants inoculated with the *Δpevd1*.1 and *Δpevd1*-Res1a strains (Fig. 5F).

### GhPR5 plays important roles in the interactions between *V. dahliae* and its hosts

**Fig. 6.**
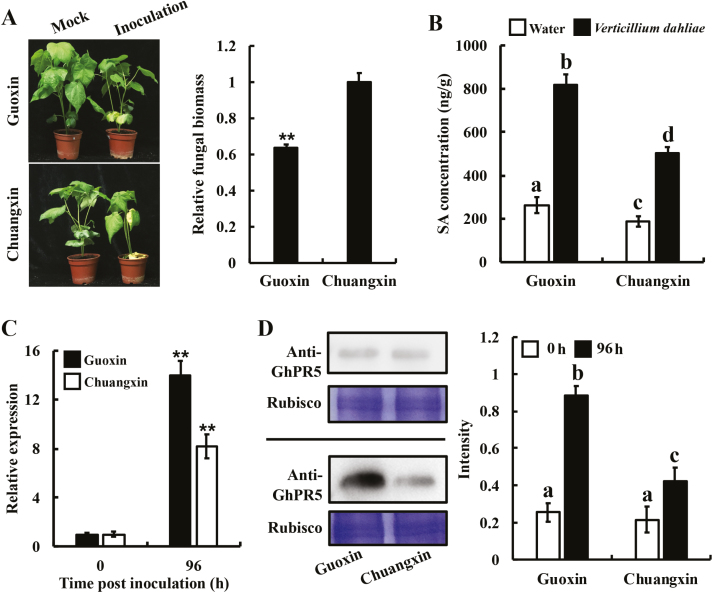
The expression of GhPR5 in two cotton cultivars, Guoxin and Chuangxin, infected with *Verticillium dahliae*. (A) Virulence was assessed using the root-dipping method with a conidial suspension (5 × 10^6^ conidia ml^–1^), and the images show symptoms at 21 d after inoculation. Fungal biomass of *V. dahliae* on cotton was determined by qPCR from 100 mg of ground roots, and is expressed relative to that of the wild-type. Data are means (±SD) of three replicates and a significant difference was determined using Student’s *t*-test: **P<0.01. (B) Salicylic acid (SA) content in roots measured 4 d after inoculating with a *V. dahliae* suspension or with water. Data are means (±SD) of three replicates and different letters indicate significant differences as determined by ANOVA and *post hoc* Tukey’s test: *P*<0.01. (C) Expression levels of *GhPR5* in roots following inoculation with *V. dahliae*, given as relative values compared with 0 h. Data are means (±SD) of three replicates and significant differences compared to 0 h were determined using Student’s *t*-test: **P<0.01. (D) Protein expression level of GhPR5 in the two cultivars at 0 h (top) and 96 h (bottom) after inoculation with *V. dahliae*. Coomassie brilliant blue (CBB) is shown as a total protein loading control. The intensity of staining on the western blots was used to quantify the protein expression. Data are means (±SD) of three replicates and different letters indicate significant differences as determined by ANOVA and *post hoc* Tukey’s test: *P*<0.01.

GhPR5 was identified as a target protein of PevD1 and it can interact with PevD1, suggesting that it has a significant role in the plant defence system against *V. dahliae*. Pathogenicity tests were first carried out on two different cotton cultivars with differing tolerance to *V. dahlia*, cv. Guoxin (more tolerant) and Chuangxin (less tolerant). Guoxin showed less severe disease symptoms and significantly less *in planta* fungal biomass than Chuangxin (Fig. 6A). We next tested whether the severity of the disease was related to the activation of cotton defence responses. The SA pathway is known to be important for plants to protect themselves against *V. dahliae*, and the downstream protein PR5 of the SA pathway is a determinant for resistance to *V. dahliae* (Tjamos *et al.*, 2005). We therefore determined the SA contents in the roots of the two cultivars and found that the concentrations were significantly higher at 4 d after inoculation compared with controls, and the SA concentration in Guoxin was significantly higher than that observed in Chuangxin (Fig. 6B). We also used qPCR to examine the level of *GhPR5* expression and found that the relative increase in *GhPR5* mRNA levels in Guoxin was more than that observed in Chuangxin (Fig. 6C). In addition, mRNA levels for *PR5*s were observed to increase in every host studied during *V. dahliae* infection (Supplementary Fig. S6). Given that the mRNA background level of *GhPR5* may have been different between the two cultivars, western blot assays were carried out to determine the GhPR5 protein levels. The results confirmed that the production of GhPR5 protein was induced by *V. dahliae* in both cultivars (Fig. 6D). The signal intensity of GhPR5 induced by *V. dahliae* in Guoxin was significantly higher than that in Chuangxin, which was consistent with the results for SA content. These data suggest that GhPR5 is an important determinant for cotton resistance against *V. dahliae*.

### PevD1 protects *V. dahliae* against the antifungal activity of GhPR5

**Fig. 7.**
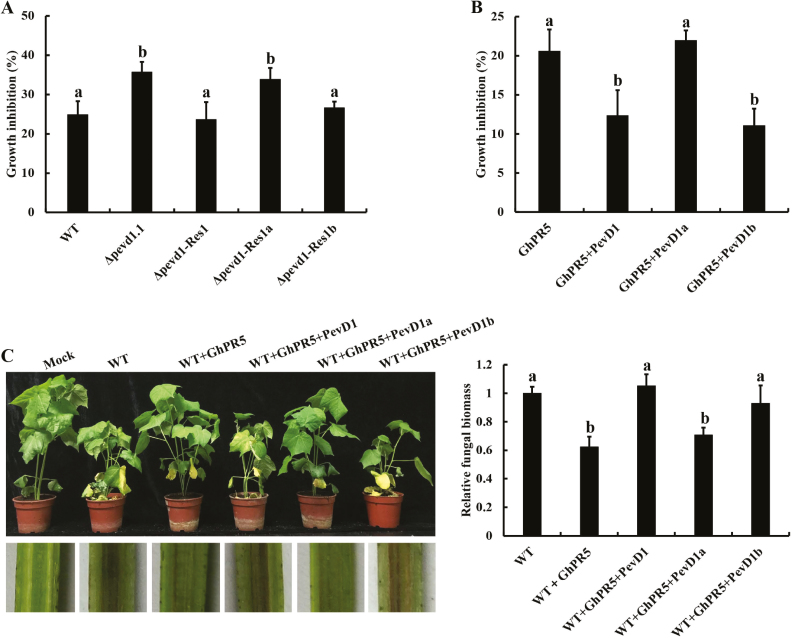
PevD1b protects *Verticillium dahliae* against the antifungal activity of GhPR5. (A) The sensitivity of different *V. dahliae* strains to the presence of GhPR5; WT, wild-type. Growth was determined spectrophotometrically by measuring the absorbance of the resazurin vital dye at 578 nm. Data are means (±SD) of three replicates and different letters indicate significant differences as determined by ANOVA and *post hoc* Tukey’s test: *P*<0.01. (B) The sensitivity of the *V. dahliae* WT strain to the presence of GhPR5 with or without PevD1 (and its truncated fragments). Data are means (±SD) of three replicates and different letters indicate significant differences as determined by ANOVA and *post hoc* Tukey’s test: *P*<0.01. (C) Representative images showing symptoms in cotton plants (*G. hirsutum* cv. Chuangxin) at 21 d after being inoculated with the WT strain pre-incubated with different protein solutions, together with qPCR analysis of fungal biomass from 100 mg of ground roots. Data are means (±SD) of three replicates and different letters indicate significant differences as determined by ANOVA and *post hoc* Tukey’s test: *P*<0.01.

We tested the sensitivity of different *V. dahliae* strains to the presence of GhPR5 and found that the growth of *Δpevd1*.1 and *Δpevd1*-Res1a were significantly inhibited compared to the WT, *Δpevd1*-Res1, and *Δpevd1*-Res1b strains (Fig. 7A), suggesting that PevD1b plays a crucial role in protecting *V. dahliae* against GhPR5. A growth-inhibition assay of the WT strain was also performed using different protein PevD1 constructs, which indicated that PevD1b but not PevD1a decreased the antifungal activity of GhPR5, although the inhibition was not complete (Fig. 7B). We also examined the effect of a pre-co-incubation with GhPR5 on *V. dahliae* virulence in which cotton plants were inoculated with *V. dahliae* WT conidia that had been germinated with GhPR5 (2 μM) or with GhPR5 plus a PevD1 protein for 12 h. The virulence of *V. dahliae* incubated with GhPR5 alone was significantly reduced compared to that of *V. dahliae* incubated with GhPR5 and PevD1 or PevD1b (Fig. 7C), indicating that PevD1/PevD1b could protect *V. dahliae* by inhibiting the antifungal activity of GhPR5.

## Discussion

Among the vast array of proteins secreted by *V. dahliae*, one of the most abundant is PevD1 (Wang *et al.*, 2012). Our previous studies indicated that PevD1 could induce plant defence responses (Wang *et al.*, 2012) and interact with NRP1 proteins, which mediated early flowering in Arabidopsis (Zhou *et al.*, 2017) and disease resistance in tobacco (Liang *et al.*, 2018). As the only Aa1-like protein in *V. dahliae*, the pathogenic function of PevD1 during infection has remained elusive. In the present study, we first found that PevD1 contributes to *V. dahliae* pathogenicity in cotton via its interaction with the protein GhPR5. The mRNA levels of *pevd1* showed up-regulation during the early stages of infection in a variety of hosts (Fig. 2C). Although the phenotypes of the *Δpevd1* strains were not discernibly different from those of the WT strain with respect to their growth rate, sporulation capacity, and mycelium/spore morphology (Fig. 2D–F), they displayed significant decreases in their virulence against hosts, and complementation of *pevd1* resulted in restoration of virulence to the levels observed for the WT strain (Fig. 2G). To date, few studies have investigated whether AA1 family proteins have roles in plant pathogenesis. Mitakakis *et al.* (2001) showed that high amounts of Aa1 are released in germinating compared to ungerminated conidia, corresponding to our qPCR results for *pevd1* (Fig. 2C). These data suggest that PevD1 plays an important role in the pathogenicity of *V. dahliae*.

We formed a hypothesis that PevD1 can act as an effector to contribute to *V. dahliae* infection, and thus we attempted to identify the interacting protein partner of PevD1 in cotton using pull-down assays. A PR5-like protein was isolated and was designated as GhPR5. Many investigations have shown that PR5s can be induced by pathogen attacks (e.g. Anil Kumar *et al.*, 2015). One of the best-characterized properties of PR5s is their antimicrobial activity against a wide variety of organisms, including *B. cinerea*, *P. infestans*, *V. dahliae*, *V. alboatrum*, and *F. oxysporum*, and overexpressing a PR5-like protein in plants can enhance their resistance to pathogenic fungi (Anil Kumar *et al.*, 2015). AtPR5 in Arabidopsis has been reported to show a stronger induction after inoculation with *V. dahliae* and is considered as a determinant of resistance (Tjamos *et al.*, 2005). In our present study, the results showed that GhPR5 was markedly induced by *V. dahliae* infection (Fig. 6D) and possessed anti-fungal activity *in vitro* (Fig. 5A). Several PR5 proteins with antifungal activity have been reported to exhibit β-1,3-glucanase activity (Grenier *et al.*, 1999; Menu-Bouaouiche, 2003; van Loon *et al.*, 2006), although there is no clear evidence regarding the correlation between the two activities. Whether GhPR5 possesses β-1,3-glucanase activity requires further investigation.

Chitinase is a PR protein produced in plant cells after a pathogen infection (van Loon *et al.*, 2006) and it is able to degrade fungal cell walls into chitin oligomers that can be recognized by plant cell LysM receptors (Miya *et al.*, 2007), thus triggering the plant immunity system and protecting them from infection. Two cerato-platanin family proteins from *Fusarium graminearum* and *V. dahliae* have been observed to bind to chitin and to protect cell wall polysaccharides from chitinase degradation (Quarantin *et al.*, 2016; Zhang *et al.*, 2017*a*). MoHrip1 and Aa1, homologs of PevD1, have been shown to partially localize to the cell wall (Twaroch *et al.*, 2012; Zhang *et al.*, 2017*b*), and therefore we suggest that PevD1 may become enriched in cell walls to protect them from being degraded by GhPR5. As shown in the present study, GhPR5 was more active against *Δpevd1* mutants than against the WT strain (Fig. 7), and the anti-*V. dahliae* activity of GhPR5 was decreased *in vitro* by the addition of PevD1, which corresponded to the results of assays showing that PevD1 contributed to virulence (Fig. 2G). Fungal cell walls contain a crucial β-1,3-glucan structural component (Fesel and Zuccaro, 2016) that can act as an elicitor of plant immune responses, including SA-dependent defence (Klarzynski *et al.*, 2000). PevD1 may contribute to virulence by protecting the cell wall, thus allowing fungal growth to be maintained and preventing the recognition of degraded β-1,3-glucan by the plant.

**Fig. 8.**
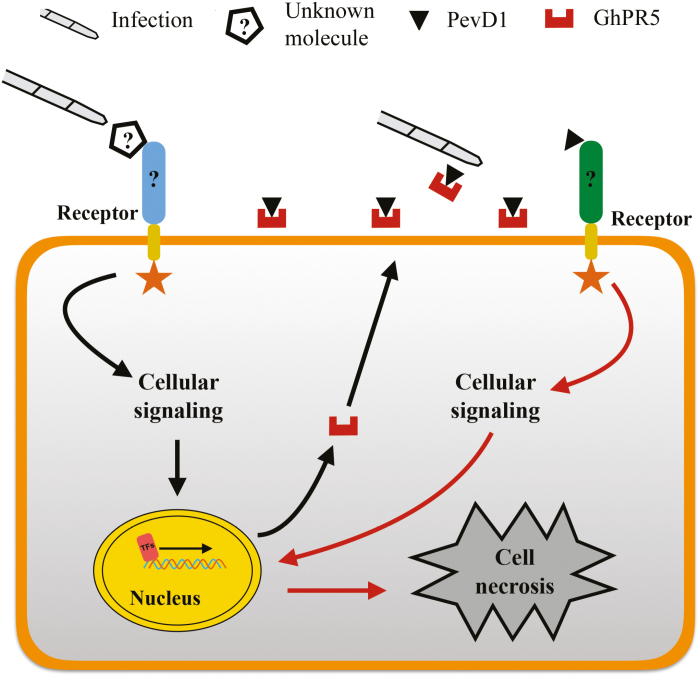
Proposed model of PevD1 function during *Verticillium dahliae*–host interaction. Upon infection, a receptor localized in the plant plasma membrane recognizes an unknown molecule of *V. dahliae*, which activates cellular signalling and results in the production of GhPR5. Secretion of PevD1 leads to an interaction with GhPR5 and protects the fungus against its antifungal activity. In addition, non-interacting fragments of PevD1 are recognized by the cell via unknown receptors and initiate cellular signalling that induces necrosis to provide nutrients for *V. dahliae* infection.

PR5s have been shown to be common host proteins involved in defence responses and possess antifungal activity against many plant pathogens. In addition to PevD1, several effectors have been previously described to interact with PR5s, such as BEC1054 from *Blumeria graminis* (Pennington *et al.*, 2016) and BcIEB1 from *B. cinerea* (González *et al.*, 2017); thus, PR5s may be targets to be attacked by pathogens. Although BcIEB1 can cause necrosis in tobacco (Frías *et al.*, 2016), its activity is disrupted by osmotin (González *et al.*, 2017). In our study, the PevD1–GhPR5 interaction resulted in a decrease in the antifungal activity of GhPR5 in cotton and promoted *V. dahliae* infection (Fig. 7). Both PevD1 and a PevD1–GhPR5 mixture could induce an equal level of necrosis in cotton and tobacco leaves (Supplementary Fig. S7), suggesting that PevD1 has complicated pathogenic roles in the infection process. In general, necrosis-inducing proteins act as virulence factors during the pathogen infection process, especially for necrotrophic fungi (Frías *et al.*, 2011; Yang *et al.*, 2017). The contribution of necrosis-inducing proteins to the virulence of necrotrophic fungi is believed to be through their ability to provide nutrients for fungal growth (Govrin and Levine, 2000; Choquer *et al.*, 2007). *Verticillium dahliae* is a hemibiotrophic fungus that produces a number of effectors that are secreted during infection to destroy plant cells (Fradin and Thomma, 2006). PevD1 was expressed at high levels from early infection onwards (Fig. 2C), suggesting that it has important roles throughout the infection process, especially in the later stages. Furthermore, PevD1 induced plant cell death in a dose-dependent manner (Supplementary Fig. S8), and it may play a phytotoxic role and promote *V. dahliae* infection by its accumulation during the late stages of infection. In summary, we suggest that the contribution of PevD1 to virulence in cotton occurs through its interaction with GhPR5 and its induction of plant cell death, which inhibit the antifungal activity of GhPR5 and provide nutrients for disease development (Fig. 8). Our results provide important insights into the strategies used by *V. dahliae* to infect hosts.

## Supplementary data

Supplementary data are available at *JXB* online.

Fig. S1. Deletion and complementation of *pevd1*.

Fig. S2. Example images showing the disease symptoms at 21 d post-inoculation in tobacco and tomato plants inoculated the *V. dahliae* wild-type, *Δpevd1*, and *Δpevd1*-Res1 strains compared with a mock inoculation.

Fig. S3. The expression and purification of PevD1 and its truncated fragments.

Fig. S4. The expression and purification of GhPR5 and tag peptides.

Fig. S5. Complementation of *pevd1* fragments.

Fig. S6. Expression levels of *PR5*s in Arabidopsis, tobacco, and tomato plants inoculated with *V. dahliae*.

Fig. S7. GhPR5 does not interfere with the necrosis-inducing activity of PevD1.

Fig. S8. Assays of the necrosis-inducing ability of PevD1.

Table S1. List of primers used in this study.

## Supplementary Material

Supplementary Figures

Supplementary Table
