# Supplementary material for: Verticillium dahliae PevD1, an Alt a 1-like protein, targets cotton PR5-like protein and promotes fungal infection
Source: J Exp Bot. 2018 Oct 5;70(2):613–26. doi: 10.1093/jxb/ery351 (PMC6322577; doi:10.1093/jxb/ery351)
Supplement: Supplementary Figures [file ery351_suppl_supplementary_figures_s1-s8.pdf]

Supplementary Figure S1

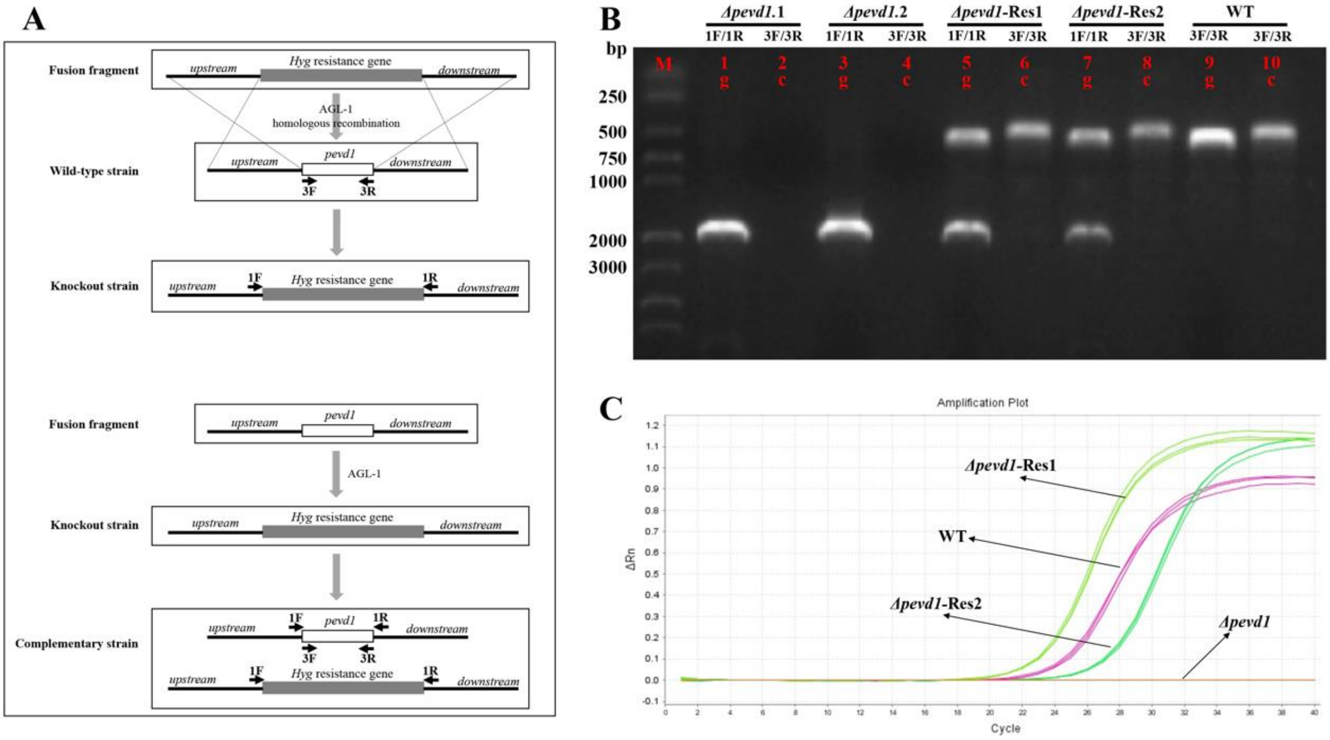

Fig. S1. Deletion and complementation of *pevd1*. (A) Schematic map of the generation of the *pevd1* knockout mutants (up) and their complementary strains (down), respectively. (B) PCR analysis of the target transformants that were used for further experiments. For the detection of Wild-type (WT), *Δpevd1* and *Δpevd1*-Res strains, extracted genomic DNA (g) and synthesized cDNA (c) were used as templates for PCR detection with the primers 1F/1R and 3F/3R, respectively. Primers 1F/1R were designed from the 20-bp 5′ region and the 20-bp 3′ region of *pevd1*, respectively. Primers 3F/3R were designed from the 5′ and 3′ region of *pevd1* mRNA sequence. M, marker. 1-10 correspond to the lanes. (C) qPCR analysis of *pevd1* gene from *V. dahliae* WT, *Δpevd1* and *Δpevd1*-Res strains.

Supplementary Figure S2

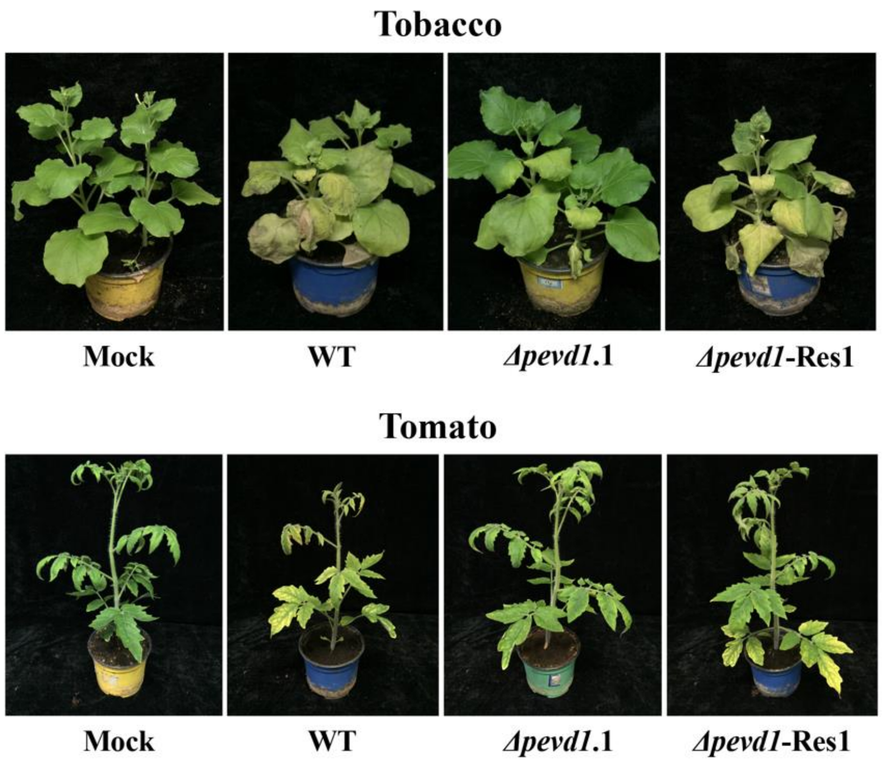

Fig. S2. The example photographs show symptoms in tobacco and tomato plants inoculated with the WT, *Δpevd1*.1 and *Δpevd1*-Res1 strains.

Supplementary Figure S3

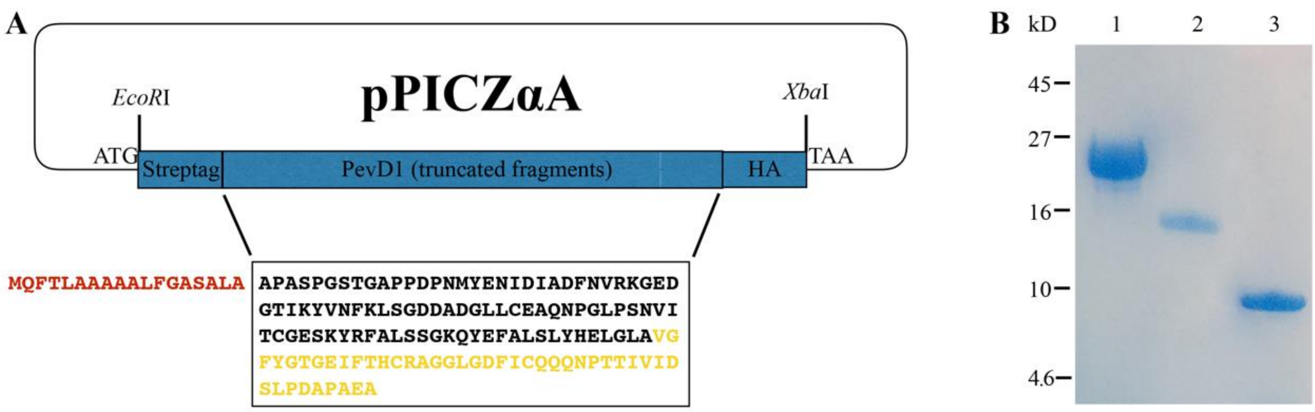

Fig. S3. The expression and purification of PevD1. (A) Map of plasmid pPICZαA-PevD1. The *pevd1* gene was cloned into the *EcoR*I and *Xba*I restriction sites of plasmid pPICZαA to generate the plasmid pPICZαA-PevD1. The recombinant plasmid contains a Streptag, HA tag and the *pevd1* gene. The N-terminal signal peptide (red sequence) is shown beside the frame. The black and yellow fragments of PevD1 correspond to PevD1a and PevD1b, respectively. (B) Detection of purified proteins by SDS-PAGE. Lanes 1, 2 and 3 show PevD1, PevD1a and PevD1b expressed by *Pichia pastoris* KM71H, respectively. The proteins were stained with Coomassie Brilliant Blue.

Supplementary Figure S4

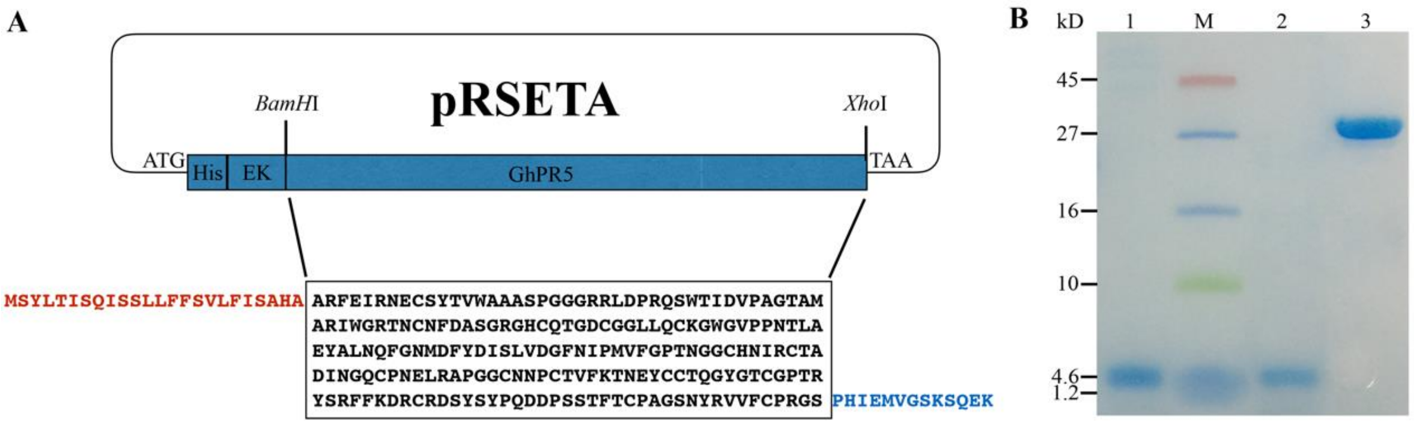

Fig. S4. The expression and purification of targeted protein/peptides. (A) Map of plasmid pRSETA-GhPR5. The *GhPR5* gene was cloned into the *BamH*I and *Xho*I restriction sites of plasmid pRSETA to generate the plasmid pRSETA-GhPR5. The recombinant plasmid contains a 6xHis tag, the EK cleavage site and the *GhPR5* gene. The N-terminal signal peptide (red sequence) and C-terminal vacuolar targeting sequence (blue sequence) are shown beside the frame, respectively. (B) Detection of purified protein/peptides by SDS-PAGE. M corresponds to protein molecular mass marker. Lanes 1 and 2 show the HA and His peptides expressed by *Pichia pastoris*, respectively. Lanes 3 show GhPR5 expressed by *Escherichia coli* BL21(DE3). The proteins were stained with Coomassie Brilliant Blue.

Supplementary Figure S5

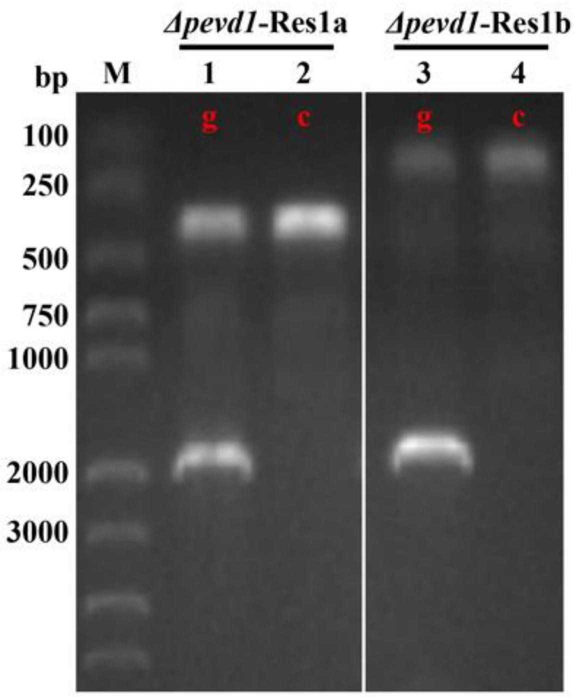

Fig. S5. Complementation of *pevd1* fragments. PCR analysis of the target transformants that were used for further experiments. M, marker. 1-4 correspond to the lanes. For the detection of *Δpevd1*-Res1a/b strains, extracted genomic DNA (g) and synthesized cDNA (c) were used as templates for PCR detection with the primers 1F/1R and 3F/3R, respectively. Primers 1F/1R (used in lanes 1 and 3) were designed from the 20-bp 5′ region and the 20-bp 3′ region of *pevd1*, respectively. Primers aF/aR (used in lanes 2) and bF/bR (used in lanes 4) were designed from the 5′ and 3′ region of *pevd1a* and *pevd1b* mRNA sequence, respectively.

Supplementary Figure S6

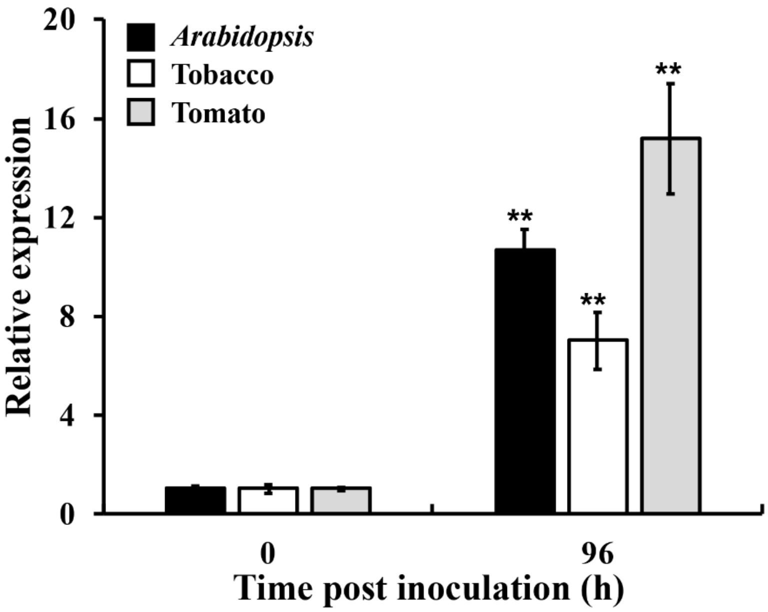

Fig. S6. Expression levels of *PR5*s in *Verticillium dahliae*-inoculated plants. The roots of *Arabidopsis*, tobacco and tomato were inoculated with *V. dahliae* spore suspension, respectively. Roots were collected for qPCR analysis to check the *PR5*s mRNA levels at 96 h post inoculation. Triplicate biological replicates were used to determine the average values for quantification. Double asterisks indicate a statistically significant difference ($p < 0.01$ by Student's *t*-test).

Supplementary Figure S7

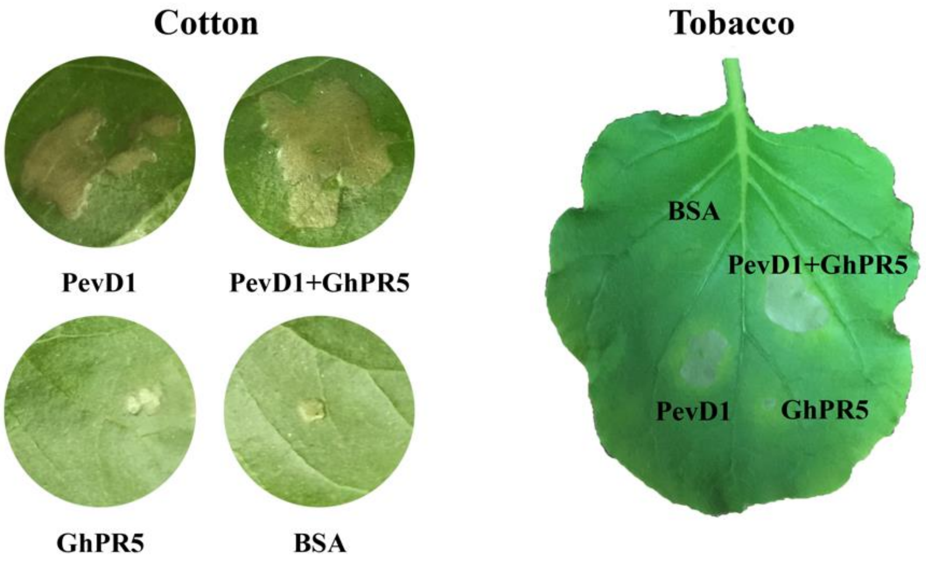

Fig. S7. GhPR5 couldn't interfere with the necrosis-inducing activity of PevD1. 20 μM of PevD1 and GhPR5 were co-infiltrated into cotton and tobacco leaves. Photographs were also taken with front illumination at 24 h post infiltration to demonstrate the effect on the leaves. PevD1, GhPR5 and BSA at the same concentration were controls.

Supplementary Figure S8

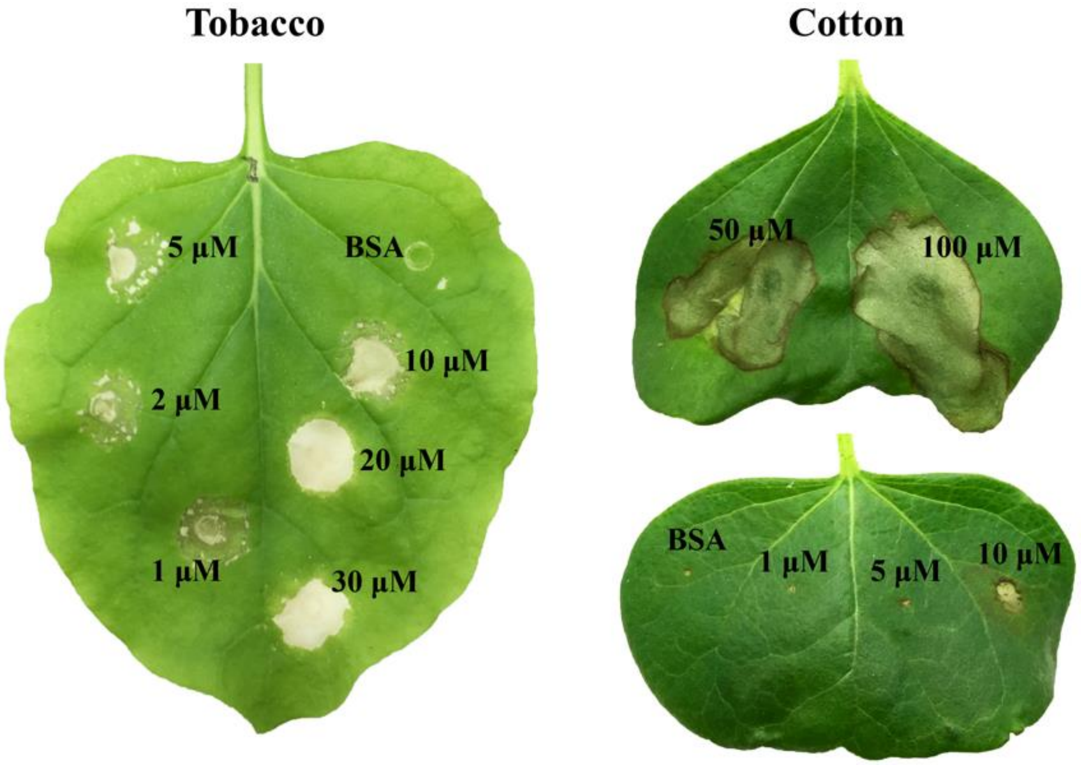

Fig. S8. Assay of the necrosis-inducing ability of PevD1. 4-week-old tobacco and 2-week-old cotton leaves were infiltrated with indicated concentration of PevD1, and images were taken with front illumination at 72 h post infiltration to assess the effect on the leaves. BSA (at the maximum concentration of PevD1) was used as control.
